# Integrative analysis of different low-light-tolerant cucumber lines in response to low-light stress

**DOI:** 10.3389/fpls.2022.1093859

**Published:** 2023-01-18

**Authors:** Dandan Li, Fushun Yu, Yanzhao Zhang, Kaihong Hu, Dongyang Dai, Siwen Song, Fan Zhang, Rina Sa, Hua Lian, Yunyan Sheng

**Affiliations:** College of Horticulture and Landscape Architecture, Heilongjiang Bayi Agriculture University, Daqing, Heilongjiang, China

**Keywords:** cucumber (*Cucumis sativus* L.), low-light stress, photosynthetic ability, gene expression, response, integrative analysis

## Abstract

**Introduction:**

Low light stress inhibits plant growth due to a line of physiological disruptions in plants, and is one of the major barriers to protected cucumber cultivation in northern China.

**Methods:**

To comprehensively understand the responses of cucumber seedlings to low-light stress, the low-light-tolerant line (M67) and The low-light-sensitive line (M14) were conducted for the analysis of photosynthetic phenotype, RNA sequencing (RNA-seq) and the expression level of photosynthesis-related genes in leaves under low-light stress and normal light condition (control).

**Results:**

The results showed that there was a sharp decrease in the photosynthate accumulation in the leaves of the sensitive line, M14, resulting in a large decrease in the photosynthetic rate (Pn) (with 31.99%) of leaves compared to that of the control, which may have been caused by damage to chloroplast ultrastructure or a decrease in chlorophyll (Chl) content. However, under the same low-light treatment, there was no large drop in the photosynthate accumulation and even no decrease in Pn and Chl content for the tolerant line, M67. Moreover, results of gene expression analysis showed that the expression level of genes *CsPsbQ* (the photosystem II oxygen-evolving enhancer protein 3 gene) and *Csgamma* (ATPase, F1 complex gene) in the M14 leaves decreased sharply (by 35.04% and 30.58%, respectively) compared with the levels in the M67 leaves, which decreased by 14.78% and 23.61%, respectively. The expression levels of genes involved in Chl synthesis and carbohydrate biosynthesis in the leaves of M14 decreased markedly after low-light treatment; in contrast, there were no sharp decreases or changes in leaves of M67.

**Discussion:**

Over all, the ability of cucumber to respond to low-light stress, as determined on the basis of the degree of damage in leaf structure and chloroplast ultrastructure, which corresponded to decreased gene expression levels and ATP phosphorylase activity, significantly differed between different low-light-tolerant lines, which was manifested as significant differences in photosynthetic capacity between them. Results of this study will be a reference for comprehensive insight into the physiological mechanism involved in the low-light tolerance of cucumber.

## 1 Introduction

Cucumber is native to the southern foothills of the Himalayas and the South Asian subcontinent (Lin, 2017). Chinese cucumber production ranks first in the world and accounts for more than 70% of the global output (Zy Consulting, 2020). High temperatures (20~32°C) during the daytime and 15~18°C at night and high light conditions (between 700 µmol·m^-2^·s^-1^ and 1000 µmol·m^-2^·s^-1^) are needed for cucumber growth (Wang, 2011). However, low-light stress is one of the major barriers to protected cucumber cultivation in northern China or other places with successive rainy and hazy weather because low light affects photosynthesis, resulting in a decrease in yield and quality potential (Liebig and Krug, 1991; Zhang et al., 2022). Under low-light stress, the growth of cucumber is inhibited, and there are several symptoms of low-light stress visible at the seedling stage. Under continuous low-light stress, the hypocoyledonary axis becomes longer, leaf chlorosis, leaf area decreases, the main axis is terminated and the shoot apical meristem converts into a flower. (Li et al., 2009), and there are no or fewer numbers of flowers and fruits, resulting in the poor cucumber quality and quantity (Ai et al., 2006; Gommers et al., 2013). Low-light stress affects the physiology of plants, such as the key enzymes involved in starch synthesis in the grains and the translocation of carbohydrates from source cells to sink cells (Du et al., 2013). Low light causes a line of physiological and biochemical disruptions in plants, and approximately 40 to 50% yield loss can occur because of low light intensity during the wet season in India and Southeast Asian countries, where a decrease in irradiation occurs 40 to 60% of the time (Venkateswarlu, 1977). It is therefore highly important to solve the above mentioned problems by studying the low-light-response mechanism and by breeding new low-light-tolerant varieties.

Continuous low-light stress can decrease the photosynthesis of leaves by disrupting photosynthetic organelles, and the effects vary among different crop species (Huang et al., 2007; Wang et al., 2009). Low-light stress can decrease the specific leaf weight and leaf thickness and can decrease the quantity and size of chloroplasts (Zhang et al., 1999; Li et al., 2021). Plants respond to and resist low-light stress *via* complex physiological changes, biochemical changes and molecular signal production (Zhou et al., 2022). The chloroplast morphology in cucumber leaves and pigment content change considerably under low-light stress (Xu et al., 2010; Kirchhoff, 2019). Chl is the most important pigment in photosynthesis and is most responsive to different environmental stresses, cucumber seedlings were shown to adapt to low-light environments *via* increased production of Chl in the leaves to capture more light during early stages of low-light stress (Zhu et al., 2012; Zhang et al., 2017). Moreover, the Chl b is responsible for transferring light energy in photosynthesis and could capture more energy to improve the utilization efficiency while contributing to the green preservation of leaves under stress (Tanaka and Tanaka, 2007; Esteban et al., 2015). The biosynthesis-related gene *HEMA1*, encoding glutamyl-tRNA reductase (GluTR), is involved in the first step of Chl synthesis, and the amount of *HEMA1* is indirectly regulated by PIF_3_ (Shin et al., 2009; Stephenson et al., 2009; Zeng et al., 2020). The chlorophyll cycle plays a crucial role in the processes of greening and acclimation to light intensity, and a certain Chl a/b ratio is needed for plants to adapt to various environments (Meguro et al., 2011). 7-Hydroxymethyl Chl a reductase (HCAR) plays critical roles in converting Chl b to Chl a when plants are under stress (Ito et al., 1996; Scheumann et al., 1998). In tomato, several genes involved in improving plant growth and alleviating photosynthetic inhibition from low-light stress have been identified (Lu et al., 2019), and physiological mechanism through which strigolactone enhances tolerance to low-light stress in cucumber seedlings has been reported (Zhou et al., 2022). Numerous studies have revealed many mechanisms underlying the shade tolerance of plants (Lichtenthaler et al., 1981; Tian et al., 2017; Ranade et al., 2019). However, the expression of genes related to Chl metabolism in different tolerant cucumber lines under low light is not clear.

Genetic studies and preliminary assessments of cucumber gene expression in response to low light have been limited to certain traits associated with low light (Li et al., 2015). The expression of photozyme- and Chl metabolism-related genes and how stress alters stomatal characteristics, decreases photosynthetic pigment contents, and disrupts the structure of photosystem II (PSII) of leaves have been thoroughly studied in various plant species (Shu et al., 2013; Sun et al., 2014; Li et al., 2021). Low light intensity strongly limits plant grain yield and quality; however, yield is not significantly reduced for some low light-tolerant lines. For example, low light does not decrease the yield of low light-tolerant rice lines (Sekhar et al., 2019). At present, the effects of low-light stress alone on cucumber plant growth, hereditary characteristics and the expression of several photosynthesis-related genes have been studied (Sun et al., 2014; Li et al., 2015; Li et al., 2019). With respect to cucumber, studies on photosynthesis and the Chl metabolic pathway and the expression of photosynthetic genes are necessary to further state the mechanism of resistance to low-light stress (Hu et al., 2021). However, there are few intensive or integrative analyses on the photosynthetic ability of tolerant and sensitive cucumber lines under low-light stress, and little information is available to elucidate the mechanism of how the tolerant cucumber lines can alleviate the effects of damage caused by low-light stress. This work therefore the photosynthetic phenotype, RNA-seq analysis and the expression of photosynthesis-related genes of leaves under semi-lethal low-light stress was conducted to explain the effects of low-light stress on photosynthetic ability and photosynthate accumulation in cucumber. The findings of this study will provide a theoretical foundation for clarifying the low-light resistance mechanism and for breeding low-light-tolerant cucumber varieties.

## 2 Materials and methods

### 2.1 Plant materials

Two homozygous cucumber inbred lines, M67 and M14, were used as plant materials. Both lines were screened by previous researchers investigating low-light tolerance. M67 is a low-light-tolerant line that grows well under low-light stress, and M14 is a low-light-sensitive line that grows abnormally with chlorotic or terminal flowering.

### 2.2 Low-light treatment and experimental design

In 2019, the plants were grown in a phytotron under a 12 h photoperiod, a mean daily temperature of 25°C/15°C (day/night), a relative humidity of 85%, and a photosynthetic photon flux density of 300 µmol·m^-2^·s^-1^ at Heilongjiang Bayi Agricultural University. The low-light treatment was applied at the two-leaf stage; the low-light intensity was 50 µmol·m^-2^·s^-1^, and the control light intensity was 300 µmol·m^-2^·s^-1^. The plants were sampled randomly at the semi-lethal time after low-light treatment with 15d for M67 and 11d for M14. The half-lethal time was setted as the days of 50% of plants stopped growing and reached a half-dead state under low-light treatment. The experiment was performed in accordance with a randomized block design and was replicated 3 times with 10 plants each.

### 2.3 Leaf ultrastructural observations

The leaf ultrastructure of seedlings at the two-leaf stage was observed in the treatment and control groups. Samples were taken from the middle position of the leaves; cut into diamonds of 5~7 mm×3~5 mm; fixed in 2.5% glutaraldehyde solution at a pH of 6.8; chilled at 4°C for 2 h; washed twice in 0.1 mol/l phosphate buffer solution; dehydrated in ethanol solution at concentrations of 50%, 70%, 95% and 100%; and transferred to a 100% acetone solution. Finally, the diamond-shaped samples were embedded in Epon 812 embedding media. After slicing and dyeing, a JEM-1200EX transmission electron microscope (Japan, JEOL company) was used for observations and imaging.

### 2.4 Determination of Chl content, photosynthetic ability and photosynthate production

The Chl content in cucumber seedling leaves was detected by the ethanol–acetone method and the content of starch and sucrose was detected by the enthrone method (Li, 2000). Furthermore, the net photosynthetic rate (Pn), stomatal conductance (Gs), intercellular CO_2_ concentration (Ci) and transpiration rate (Tr) were determined by an LI-6400 photosynthetic apparatus.

### 2.5 RNA isolation, cDNA synthesis and transcriptome analysis

In this study, samples were taken after the three low-light treatments and normal control treatments, then frozen in liquid nitrogen and stored at −80°C. Total RNA for the leaf samples was isolated using TRIzol (Invitrogen, USA), the first-strand cDNA was reverse-transcribed using the M-MLV First Strand cDNA Synthesis Kit (B532445, Sangon Biotech, Shanghai, China). The quality and concentration of the cDNA were determined using a SMA3000 UV spectrophotometer (Beijing, China). After dilution, the cDNA was stored at −20°C until use.

RNA samples that met the quality control requirements were sent to the Beijing Genomics Institute for RNA-seq analysis *via* an Illumina HiSeq 2000 (USA/Illumina). The raw data (raw reads) were filtered with the FASTQ_Quality_Filter tool from the FASTX-toolkit, which were used for further analysis. After preprocessing the RNA-Seq data, the filtered reads were subsequently mapped to the reference sequences using SOAP2 aligner/soap2 (Li et al., 2009) and were mapped to the cucumber genome database http://www.icugi.org/cgi-bin/ICuGI/genome/index.cgi?organism=cucumber (Huang et al., 2009). Differentially expressed genes were identified based on a p value ≤0.01 and | log2 ratio ≥ 1.

### 2.6 Real-time PCR analysis

Primers for PCR sequence-specific oligonucleotides were designed *via* https://www.genscript.com/tools/real-time-pcr-tagman-primer-design-tool, and *UBI* was used as an internal reference gene. The primers used for photosynthesis-related genes are listed in **Table 1**. The specificity of each pair of primers was determined by agarose gel electrophoresis and PCR product resequencing. Then, real-time PCR was performed for gene expression evaluation according to the method described by Livak and Schmittgen (2001). PCR was performed according to the instructions of the real-time PCR machine used (ABI 7500, Applied Biosystems). The PCR program was as follows: 40 cycles of denaturation (95°C for 10 min), amplification and quantification (95°C for 10 sec, 55~60°C for 30 sec, and 72°C for 30 sec per single fluorescence measurement); melting curve analysis (65~95°C, with a heating rate of 0.2°C·s^-1^ and continuous fluorescence measurements); and final cooling to 12°C.

**Table 1:** Primers of photosynthesis-related genes.

Gene symbol	Accession number	Forward primer(5’-3’)	Reverse primer(5’-3’)
*CsHemA*	*Csa7G068600.1*	GTTTGACGACGTGCTTTGCT	CGATCTCGCACTTAGGACCC
*CsHemH*	Csa2G351800.1	CCGTTTCCTCCAAGAGCCAT	ACCAATAGCGCATTCCCACA
*Csnol*	Csa3G130900.1	TCGATACTCGAACGGGCTTG	GAGGCATAACGGTAGGAGCC
*CsHemY*	Csa6G007980.1	CTGGTGTTAGTGGGCTTGCT	CCTCCAACTCTTTCGTCTGCT
*CsCAO*	Csa6G385090.1	AACCGTATCATCCCCGCTTG	AAGGACATTGGACTCGACCG
*PsbQ*	Csa3G414060.1	TTCTCCGCCATTCCCAATCTC	TGCATTGCCAAAGAGAGCAAC
*Csgamma*	Csa6G513760.1	CTTTCCGGCCAGTCAATCCT	CGATCCGTTCTCGAAGCTCA
*CsSUS2-1*	Csa4G001950.1	GGAGGACAGTGAAGGGTTGG	AGGATCGGATTGGACGAGGA
*CsGBE1-1*	Csa6G357030.1	GGAGGACAGTGAAGGGTTGG	TCATCTTCGGGTTTGCTCGT
*Cssps*	Csa2G401440.1	CTAGCTGGCCTCCACAAGAC	GCCAAAAGATCATGGACGCC
*CsUGP2*	Csa3G307690.1	CCAGAGCACTTCCCTTCGTT	GAGAACGACACCAGACCCAA
*CsglgA-1*	Csa1G062920.1	TCTGGTTCGGCAAAGTGGAA	ACGGGTGCACTAGACCAATC
CsBam6-3	Csa6G072990.1	CCAGAGCACTTCCCTTCGTT	ACATGGTGTCTCGGCAATGT

### 2.7 Statistical analysis

The data were analyzed by analysis of variance (ANOVA) using the SPSS 13.0 statistics program, and statistical significance among the differences was calculated by one-way ANOVA followed by Duncan’s multiple range tests for each experiment at a level of *P <* 0.05. The Origin 8.0 data are expressed as the mean values ± standard deviations of three independent experiments (*n* = 3). Quantitative real-time polymerase chain reaction (qRT–PCR) analysis was performed according to Rao et al. (2013) methods.

## 3 Results

### 3.1 Morphological observations of different low-light-tolerant cucumber lines

**Figure 1:**
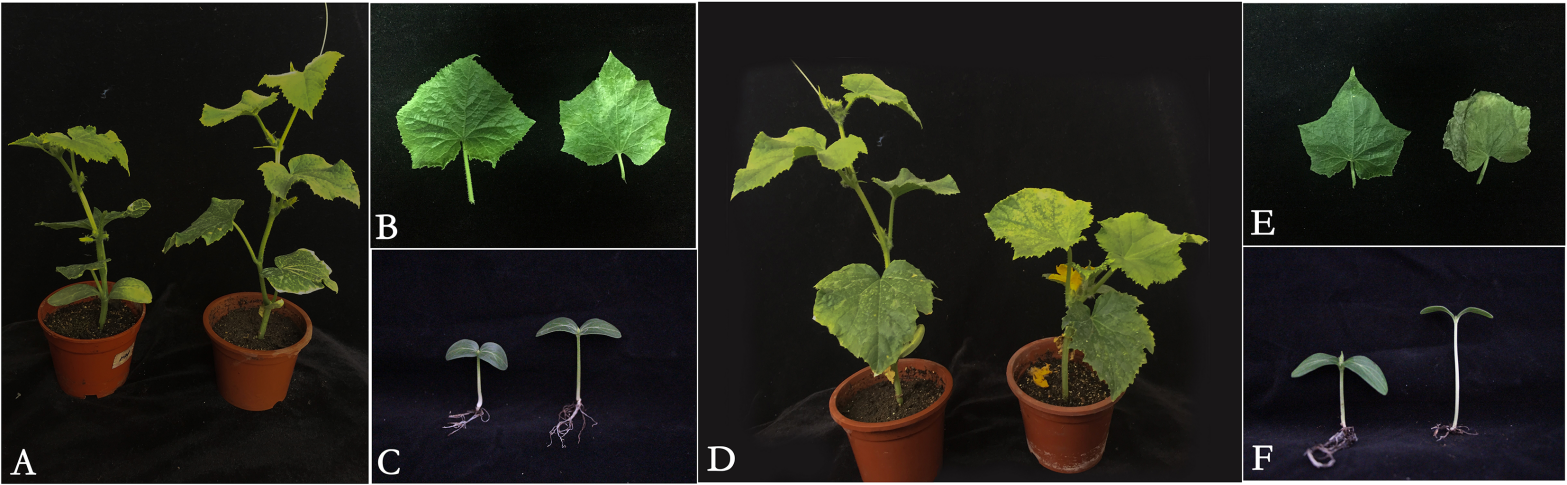
Seedling of cucumber lines with different tolerance under low-light stress. **(A)** The plant of low-light-tolerant line M67 under normal light and semilethal low- light stress (from left to right), **(B)** The leaf of low-light-tolerant line M67 seedling with two leaves under normal light and semilethal low-light stress (from left to right), **(C)** The hypocotyl of low-light-tolerant line M67 seedling in cotyledonous stage under normal light and semilethal low-light stress (from left to right), **(D)**The plant of low-light-sensitive line M14 under normal light and semilethal low-light stress (from left to right), **(E)** The leaf of low-light sensitive line M14 seedling with two leaves under normal light and semilethal low-light stress (from left to right), **(F)** The hypocotyl of low-light-sensitive line M14 seedling in cotyledonous stage under normal light and semilethal low-light stress (from left to right).

In our previous study, the tolerance of cucumber seedlings under low-light stress was determined based on several morphological and cytological indicators (Li et al., 2009; Li et al., 2015; Zhang et al., 2022). In this study, the morphological and cytological structures in cucumber leaves of seedlings at the tow-leaf stage under low-light treatment were observed from 0 to 15 days of treatment. M67 (an cucumber inbred line with a high low-light-tolerance index) was used as a low-light-tolerant line, and M14 (an inbred line with a small low-light-tolerance index) was used as a low-light-sensitive line (**Figure S1**). The index of low-light tolerance of the tolerant line (M67) was 2.39, which was higher than that of the sensitive line (M14), with an index of 1.48; thus, there was a significant difference of 38.09% between the two lines (**Supplementary Table 1**). The growth of tolerant and sensitive (M67 and M14) seedlings was significantly different under low-light stress. Morphological observations revealed that the growth of tolerant line M67 seedlings was initiated with the growth increase of internodes, there were no terminal flowering or chlorotic symptoms on the plants (**Figures 1A, B**), and the growth of hypocotyl is normal (**Figure 1C**). While under the same low-light treatment, the internode elongation rate, of the sensitive line M14 decreased, leading to the terminal flowering occurred from the 2-leaf stage to the semi-lethal point (**Figure 1D**), along with the presence of chlorosis symptoms, smaller leaf growth (**Figure 1E**) and the excessive growth of hypocotyl (**Figure 1F**).

### 3.2 Photosynthesis accumulation and photosynthetic capacity analysis in different low-light-tolerant cucumber lines

**Figure 2:**
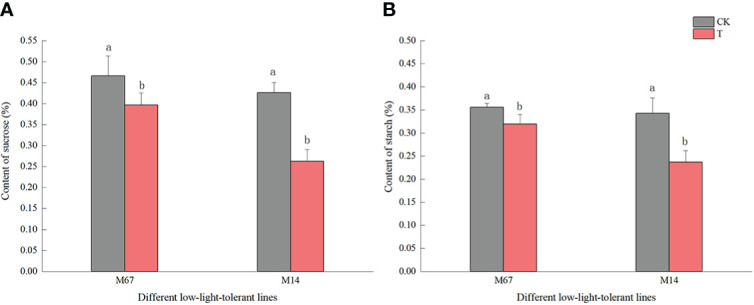
Effects on content of starch and sucrose in leaf of low-light stress in different cucumber lines. **(A)** starch and **(B)** sucrose content in leaf under control and low-light stress in different cucumber lines. Lowercase letters a and b after the value represent statistically significant differences (p < 0.05) within a variety under different low-light treatments as determined by the least significant difference test.

**Figure 3:**
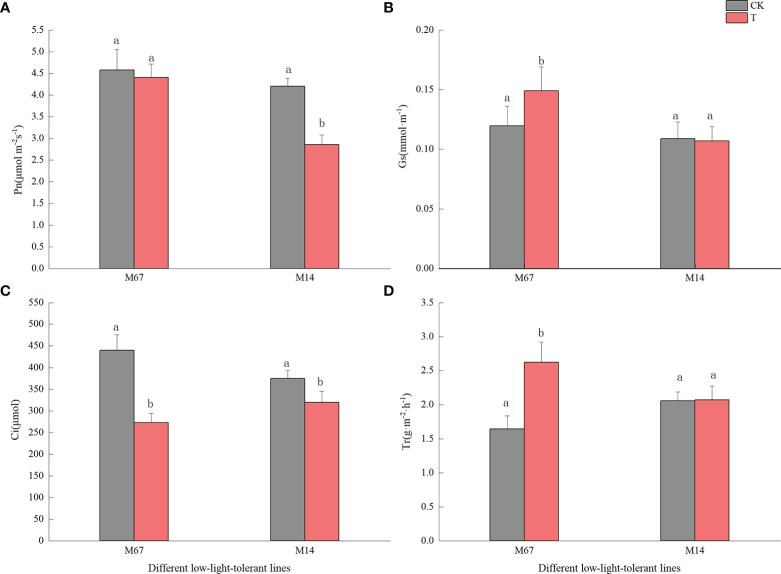
Effects on leaf photosynthetic rate of low-light in different cucumber lines. **(A)** Pn, **(B)** Gs, **(C)** Ci and **(D)** Tr of leaf under control and low-light stress in different cucumber lines. Lowercase letters a and b after the value represent statistically significant differences (p < 0.05) within a variety under different low-light treatments as determined by the least significant difference test.

**Figure 4:**
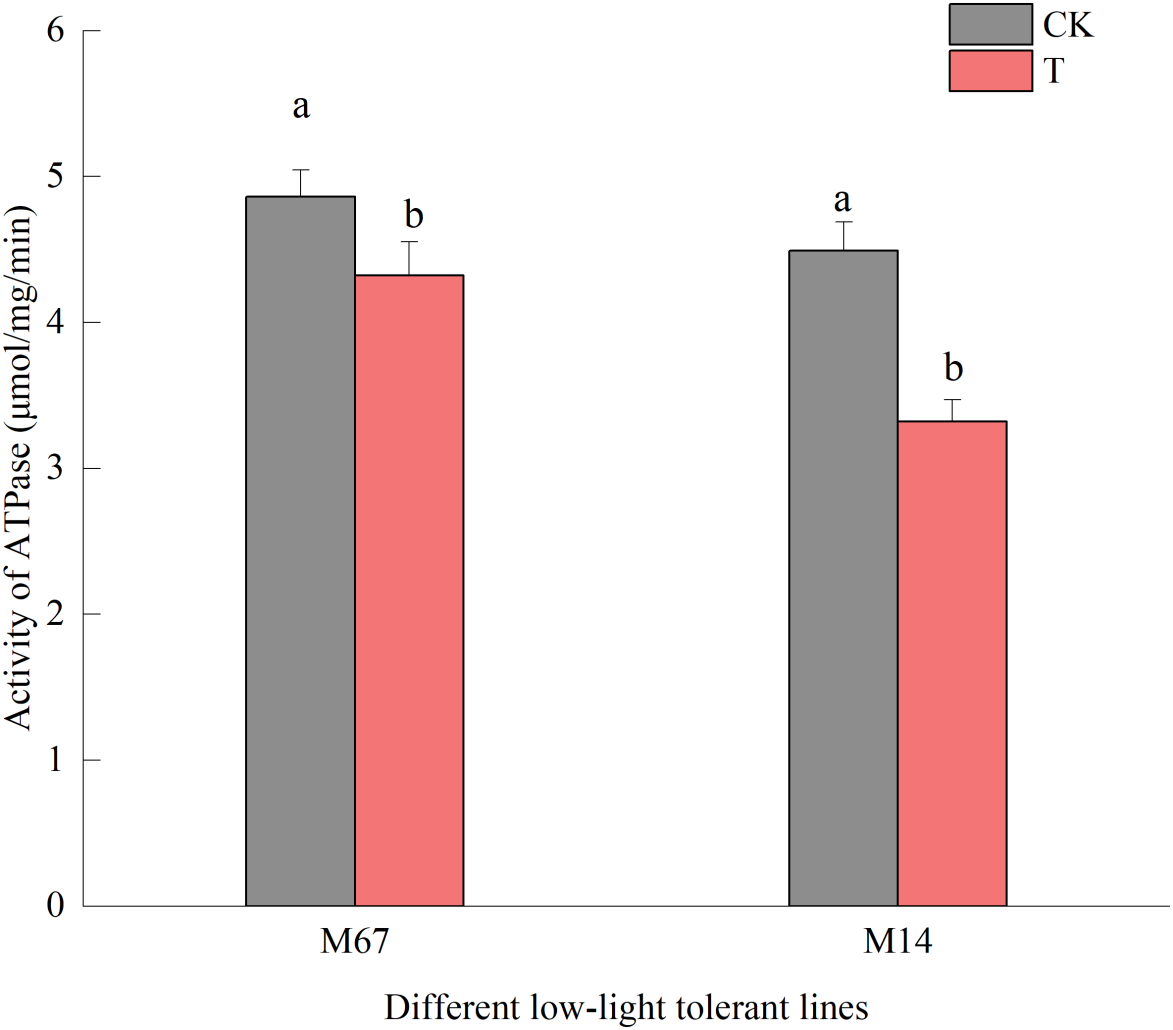
Effects on activity of ATPase of low light in different cucumber lines. Lowercase letters a and b after the value represent statistically significant differences (p < 0.05) within a variety under different low-light treatments as determined by the least significant difference test.

Compared to the normal light, the contents of starch and sucrose in cucumber seedling leaves decreased to a varying degrees under the low-light stress. After the low-light treatment, the contents of sucrose and starch in leaves of the M67 line decreased by 12.03% and 10.19%, respectively, and larger decreases of 38.36% and 30.76% in starch and sucrose contents were detected in M14 (**Figure 2** and **Supplementary Tables 2**, **3**), which indicated a sharply decreased rate of photosynthate accumulation in the leaves of the sensitive line (M14). For the Pn, there was no significant decrease in the tolerant line (M67) after low-light treatment; however, there was a decrease in Pn by 31.99% in M14 under low light compared with normal light (**Figure 3A** and **Supplementary Table 4A**). For Gs and Tr, there were significant increases of 35.43% and 59.36%, respectively, in M67 after low-light treatment but no change in M14 (**Figures 3B, D** and **Supplementary Tables 4B, D**). We also found that the intercellular carbon dioxide concentration (Ci) of the tolerant line (M67) decreased by 37.96%, however, that decreased only by 14.79% in M14 under low-light treatment (**Figure 3C** and **Supplementary Table 4C**). The data in **Figure 4** show that the ATP phosphorylase activity in the leaves of cucumber seedlings under semi-lethal treatment was lower than that under normal-light conditions, and the decreases were 27.63% and 32.26% for the tolerant and sensitive lines, respectively (**Supplementary Table 5**).

### 3.3 Chloroplast ultrastructure and pigment content observations in different low-light-tolerant cucumber lines under low-light stress

**Figure 5:**
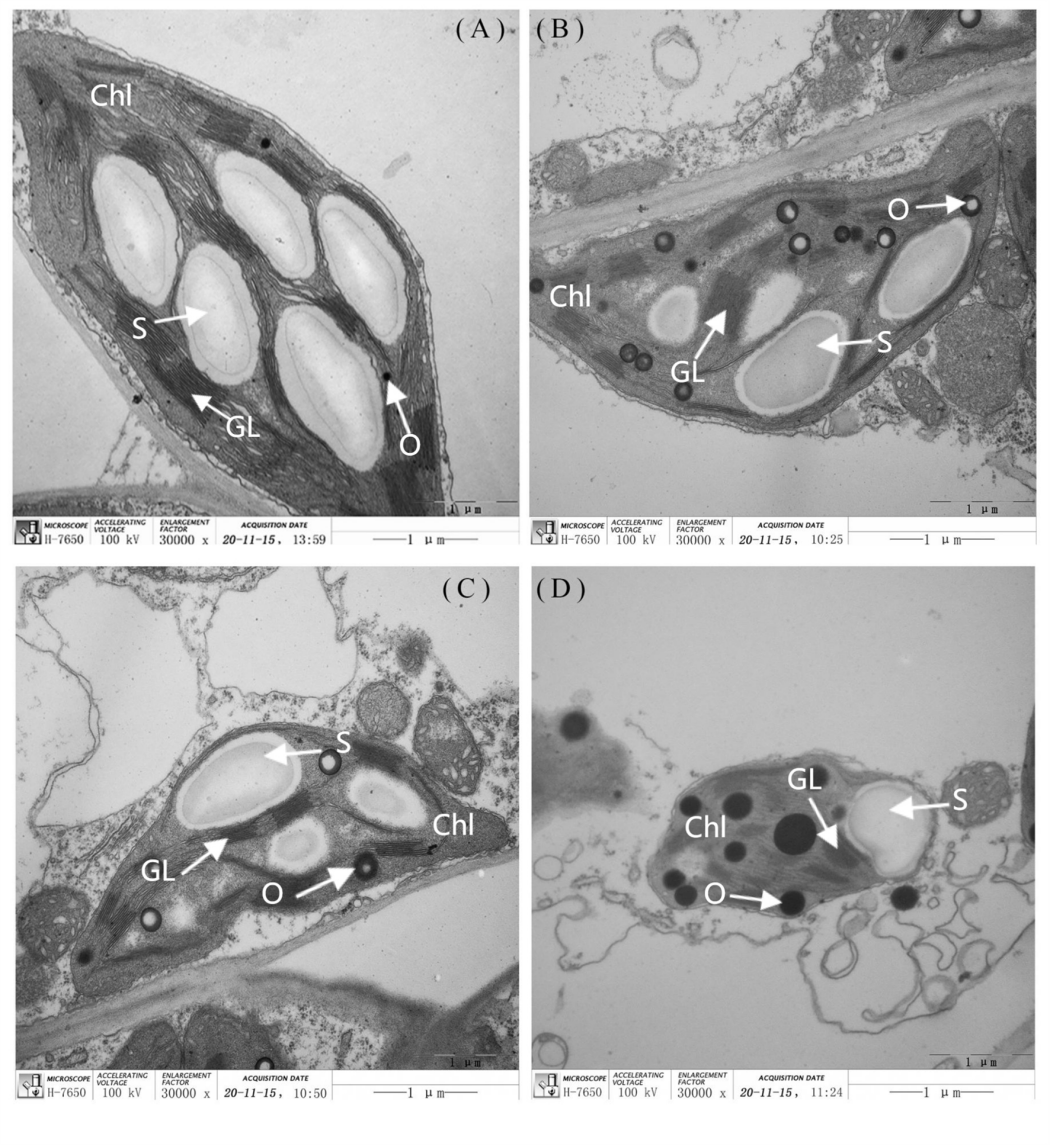
The ultra-structure of leaf chloroplast observation in different cucumber lines. The **(A)** and **(C)** are each representing the mincrostructure of chloroplast (×30000) in M67 and M14 under normal light, the **(B)** and **(D)** are each representing the mincrostructure of chloroplast (×30000) in M67 and M14 in low light treatment. Chl, Chloroplast; S, Starch; GL, Grana lamella; O, Osmiophilic globules.

**Figure 6:**
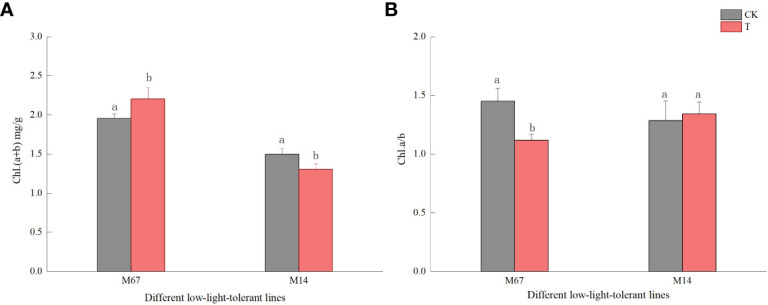
Effect on chlorophyll content in leaf of low light in different cucumber lines. **(A)** Chl (a+b) content and **(B)** Chl a/b of leaf in different cucumber lines. Lowercase letters a and b after the value represent statistically significant differences (p < 0.05) within a variety under different low-light treatments as determined by the least significant difference test.

The leaf chloroplast ultrastructure observations indicated that the chloroplasts were closely arranged near the cell wall and presented a complete structure that was filled with many starch grains in the matrix and thylakoids under normal-light conditions. The lamellae of the matrix and thylakoids were clearly visible and orderly packed (**Figures 5A**, **C**). The structure and shape of the chloroplasts of inbred line M67 were still intact and regularly spindle-shaped, and the density of the matrix and thylakoids decreased under low-light stress (**Figure 5B**). However, most misaligned chloroplasts in the sensitive line (M14) pulled away from the cell wall towards the middle, the membrane structure was severely damaged, and the lamina was cavitated and close to rupturing. The mesophyll cells had few starch grains, and there were more osmiophilic cells in M14 than in M67 (**Figure 5D**). Observations of Chl contents in the different low-light-tolerant cucumber inbred lines under low-light stress revealed that the changes in the total Chl content in the functional leaves under semi-lethal low-light stress were different; the content of Chl (a+b) in the leaves of the M67 inbred line increased by 12.67%, which was significantly higher, and the content of Chl (a+b) in the leaves of the M14 lines decreased extremely significantly – by 13.05% (**Figure 6A** and **Supplementary Table 6**). Under low-light stress, the ratio of chlorophyll a to b (Chl a/b) in the leaves of the tolerant line (M67) was 22.92% lower than that under normal-light conditions, and the Chl a/b of the sensitive line (M14) did not substantially change under either light condition (**Figure 6B** and **Supplementary Table 7**).

### 3.4 Transcriptome and photosynthesis-related metabolic pathway analysis of cucumber leaves with semilethal effects under low-light stress

**Figure 7:**
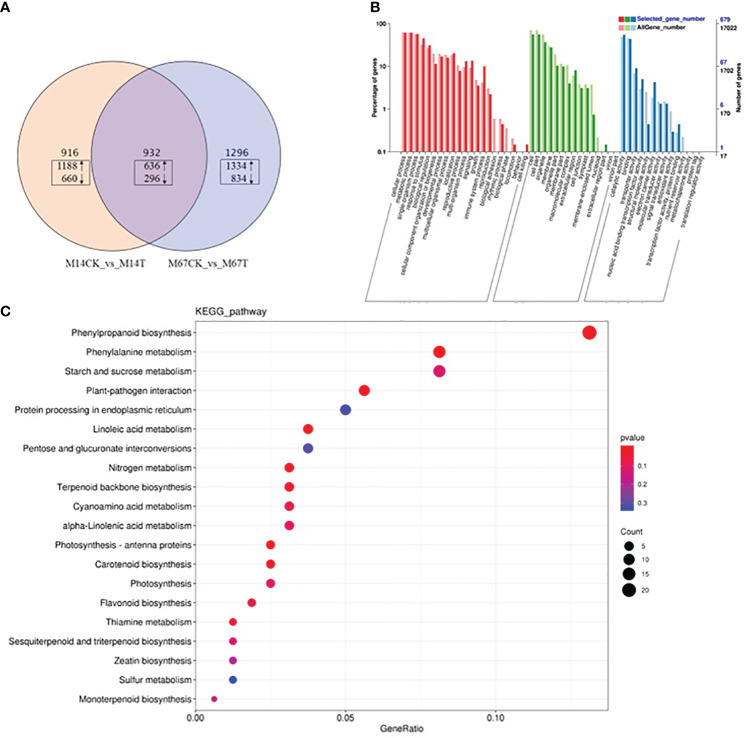
Transcriptome analysis of cucumber leaves with semilethal in M67 and M14 under low-light stress. M67: tolerant line. M14: sensitive line. **(A)** Venn diagram of DEGs under low-light. The DEGs sets (M14CK Vs M14T and M67CK Vs M67T) were analyzed by using the Venn method and the numbers in box marked in the diagram indicate the number of common genes significantly up- (upward arrows) and down-regulated (downward arrows) among the three DEG sets (log2-fold change ≥1.5 and FDR-corrected P-value ≤0.001). **(B)** GO enrichment analysis result of 932 DEGs with fold change >2. **(C)** KEGG enrichment of 932 DEGs. The bubble diagram shows the degree of enrichment of KEGG terms in three categories. By default, the top 20 GO terms with the lowest Q-values were used in the diagram. The X-axis represents the enrichment ratio, and the Y-axis denotes the KEGG pathway. The size of bubbles indicates the number of genes annotated to a certain KEGG pathway, and the color represents the Q-value, where the redder the color is, the smaller the Q-value is.

**Figure 8:**
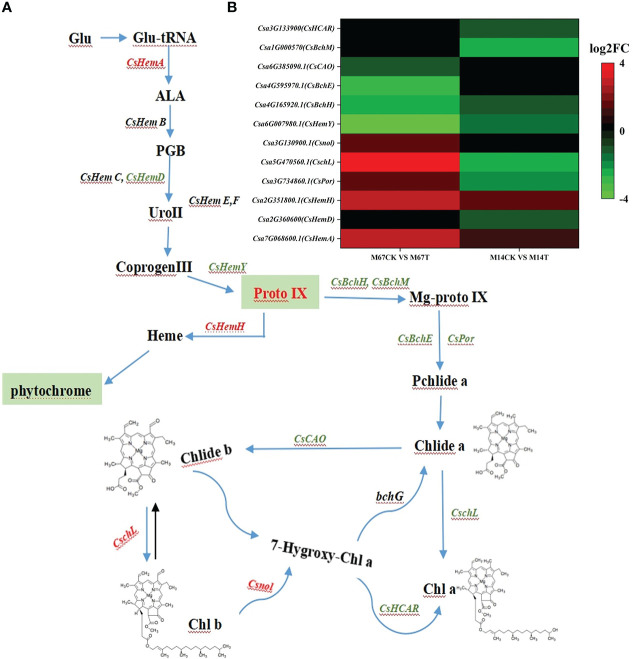
**(A)** Pathway of porphyrin and chlorophyll metabolism, and green letters were downregulated expression genes and red letters represented upregulated expression genes. **(B)** The Expression patterns of the differently expressed genes (DEGs) induced by low-light stress in the pathway of porphyrin and chlorophyll metabolism pathway. The expression levels were calculated in fragment per kilobase per million reads (FPKM) from triplicate experiments. The heat map shows the normalized value Z-score after log2 (FPKM) transformation. The color in the heat map represents the Z-score after the transformation of log2 (mean FPKM). Each row in the heat map represents the levels of a DEG under different conditions. The DEG name and the gene ID in parenthesis were listed on the right side of the heat map.

To explore the genes differentially expressed in response to low light compared to the control conditions in both inbred lines (M67 and M14), RNA-seq was performed on leaves (15 days after low-light treatment and 15 days of normal-light treatment as control). Statistical analysis was conducted to summarize the number of clean reads that were aligned to the reference genes, which provided general information for the project. Here, 21.42 to 24.87 million paired-end reads were generated per sample (**Supplementary Table 8**). Analysis of these data sets showed that the remaining 2228 genes were differentially expressed (1394 upregulated and 834 downregulated genes) in the leaves of M67 under the control and low-light conditions, and a total of 1848 genes were differentially expressed (1188 upregulated and 660 downregulated genes) in the leaves of M14 under the control and low-light conditions (**Figure 7A**; **Supplementary Table 9**). A total of 932 differentially expressed genes (DEGs) were obtained, namely, 636 upregulated genes and 296 downregulated genes, in both the M67 and M14 lines (**Figure 7A** and **Supplementary Table 9**). Based on Gene Ontology (GO) analysis and Kyoto Encyclopedia of Genes and Genomes (KEGG) pathway enrichment of these 932 DEGs, the biological process, cellular component and molecular function significantly enriched in DEGs were detected compared with those in the whole-genome background (**Figure 7B**). Finally, the DEGs corresponding to molecular function were filtered (**Figure S3**), pathways of the photosynthesis and photosynthesis-antenna proteins significantly enriched in DEGs (**Figure 7C**). So we selected fifty-five genes related to photosynthesis enriched in the leaves, which were differentially expressed under low-light stress in the differently tolerant lines M67 and M14 (**Figure S4** and **Supplementary Table 10-2**). All above analysis were performed using BMKCloud (www.biocloud.net). Moreover, KEGG pathway analysis of photosynthesis showed that the photosystem and cytochrome b6/f complex were affected differently in both lines after low-light treatment(**Figure S2**). For M67, the *CsPsbQ*, *CsPsbW* and *CsPsbP* genes of PSII, *Csgamma* (ATPase, F1 complex gene) of PSI and *CsLhcb2* of light-harvesting protein complex were upregulated, PSII genes *CsPsbB*, *CsPsbS* and more than half of complex II chl a/b binding protein (LHC) genes were downregulated after semi-lethal low-light treatment (**Figures S2A, C** and **Supplementary Table 11**). However, for the sensitive line (M14), there were no upregulated genes, and mostly genes of PSII and LHC were downregulated (**Figures S2B, D**) under the same treatment. In addition, some differentially expressed genes were detected by analysis of the Chl metabolism pathway, such as genes involved in the pathway of L-glutamyl-tRNA to Proto IX (*CsHemA*, *CsHemD and CsHemY*), some genes involved in the biosynthesis from Proto IX to Chlide a (*CsBchH*, *CsBchM*, *CsBchE* and *CsPor*), and the Chl cycle genes (*CsCAO*, *CschL*, *Csnol* and *CsHCAR*) (**Figure 8A** and **Supplementary Table 12**). The results showed that the genes *CsHemA*, *CsHemD*, *CsPor*, *CschL Csnol*, and *CsHemH* were upregulated in the tolerant line (M67) after semi-lethal low-light treatment; however, most of the genes were downregulated in the sensitive line (M14) (**Figure 8B** and **Supplementary Table 13**).

**Figure 9:**
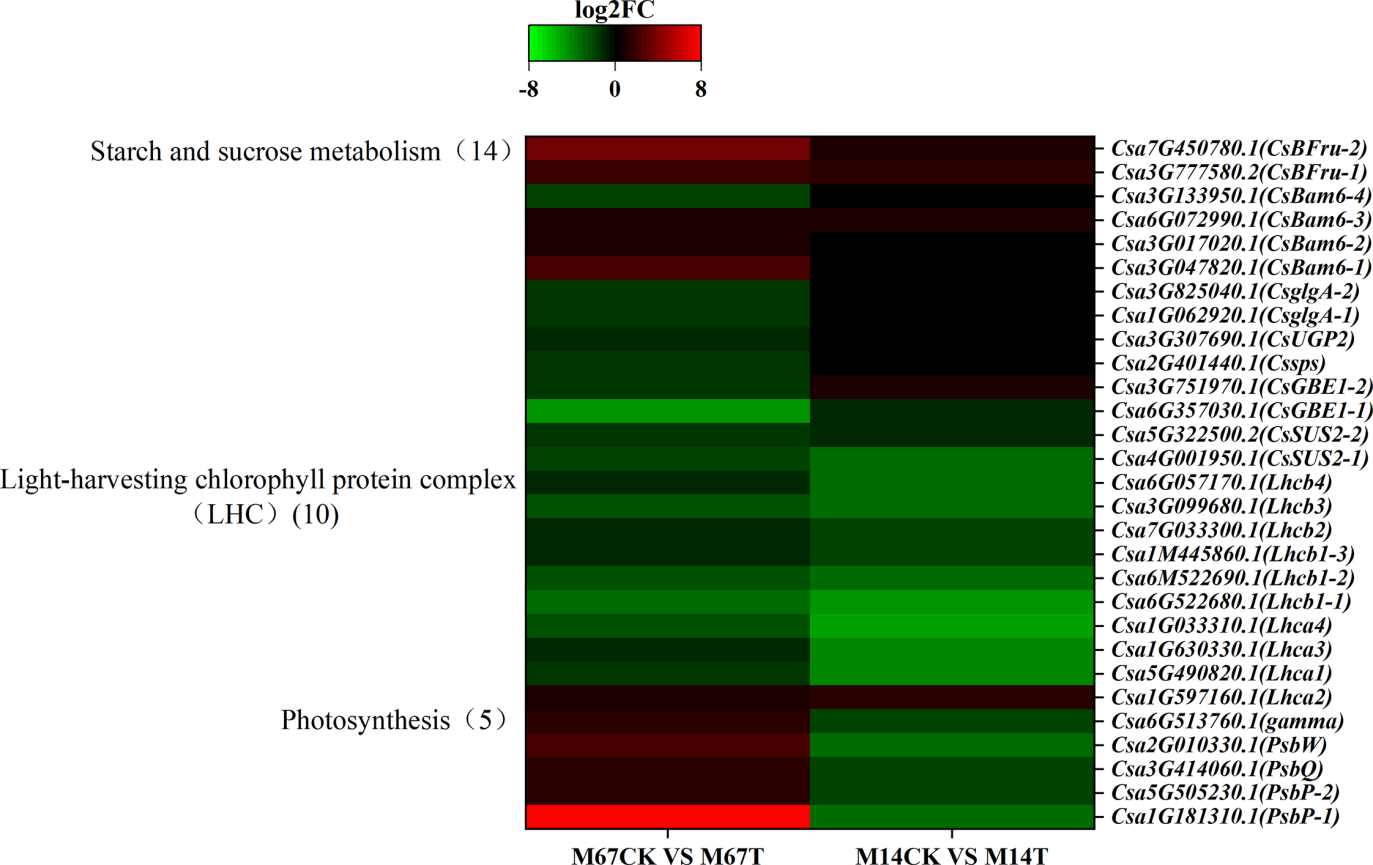
The Expression patterns of the differentially expressed genes (DEGs) induced by low-light stress in the pathway of starch and sucrose metabolism, LHC and photosynthesis. Expression change of the DEGs with at least twofold changes and p < 0.05 in this three pathways. The expression levels were calculated in fragment per kilobase per million reads (FPKM) from triplicate experiments. The heat map shows the normalized value Z-score after log2 (FPKM) transformation. The color in the heat map represents the Z-score after the transformation of log2 (mean FPKM). Each row in the heat map represents the levels of a DEG under different conditions. The DEG name and the gene ID in parenthesis were listed on the right side of the heat map.

KEGG pathway analysis of the polysaccharides revealed that the expression of starch-, sucrose- and glycogen synthesis-associated genes was dramatically different in cucumber leaves that were under semi-lethal low-light stress than in leaves under normal-light conditions. A total of 14 genes related to the starch and sugar metabolism pathway were significantly differently expressed; of these, 9 were downregulated, and 5 were upregulated in M67 leaves after low-light stress; however, only 7 genes were expressed differently in M14 (4 were upregulated and 3 were downregulated) (**Figure 9** and **Supplementary Table 13**). These genes included the sucrose synthase genes *CsSUS2s* and *Cssps*, which control UDP-glucose to sucrose and sucrose-6´P, respectively, and the gene *CsUGP2*, which controls glucose-1-phosphate (a-D-glucose-1P) hydrolysis into UDP-glucose. The starch synthase *gene CsglgAs* controls ADP-glucose into amylose, and the 1,4-alpha-glucan branching enzyme-encoding gene *CsGBE1* catalyses the conversion of amylose into glycogen and starch. The beta-amylase gene *CsBam6* is required for starch breakdown, and the *CsBFru* gene encodes the beta-fructofuranosidase protein (**Supplementary Table 14**).

### 3.5 Expression of photosynthesis-related genes in leaves with semi-lethal effects under low-light stress

**Figure 10:**
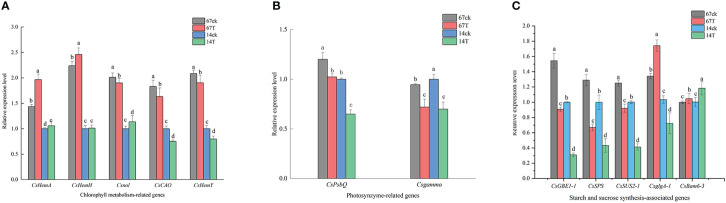
**(A)** The relative expression level of Chlorophyll metabolism-related genes. Lowercase letters a and b after the value represent statistically significant differences (p < 0.05) within a variety under different low-light treatments as determined by the least significant difference test. **(B)** The relative expression level of photosynthesis-related genes. Lowercase letters a and b after the value represent statistically significant differences (p < 0.05) within a variety under different low-light treatments as determined by the least significant difference test. **(C)** The relative expression level of starch and sucrose synthesis-related genes. Lowercase letters a and b after the value represent statistically significant differences (p < 0.05) within a variety under different low-light treatments as determined by the least significant difference test.

Based on the significant differences in phenotype and the transcription analysis results between M67 and M14 under different light conditions, the expression patterns of genes related to the Chl synthesis pathway and sucrose and starch metabolism were selected for study. For the relative expression level of genes related to Chl synthesis, different expression patterns were detected in the leaves of cucumber lines with different light tolerance after low-light treatment. The expression of genes related to Chl synthesis was detected, and we found that after low-light treatment, the expression levels of the *CsHemA* increased significantly in both lines (M67 and M14), increased by 36.91% and 5.82%, respectively. Expression levels of *Csnol* increased significantly by 13.76% in M14, but decreased by 5.49% in M67. In addition, the gene *CsHemH*, which is related to phytochrome synthesis, increased in the leaves of M67 and M14, with values of 9.99% and 1.06%, respectively.However, the expression of the *CsCAO* and *CsHemY* genes decreased in both lines, and there were smaller decrease in the leaves of M67 than in those of M14 (**Figure 10A** and **Supplementary Table 15**). The relative expression levels of *CsPsbQ* and *Csgamma* in the both lines decreased markedly after low-light treatment, and the expression levels in M14 leaves decreased sharply (by 35.04% and 30.58%, respectively) compared with the levels of *CsPsbQ* and *Csgamma* in M67 leaves, which decreased by 14.78% and 23.61%, respectively (**Figure 10B** and **Supplementary Table 16**). The expression characteristics of genes such as *CsSUS2-1*, *CsGBE-1*, *CsSPS, CsBam6-3* and *CsglgA-1* related to the starch and sucrose synthesis pathway were analysed by qRT−PCR. The results showed that except for the relative expression level of the *CsBam6-3* gene, which increased in both inbred lines after low-light treatment, the other 5 genes (*CsSUS2-1*, *CsGBE-1*, *CsUSPS* and *CsglgA-1*) decreased, with decreases of 26.34% to 69.09%, and there were larger decreases in M14 than in M67. The relative expression level of the glycoside hydrolase gene *CsBam6-3* increased by 4.85% and 17.96% in the leaves of M67 and M14, respectively (**Figure 10C** and **Supplementary Table 17**).

## 4 Discussion

In the present study, global transcriptional events of cucumber leaves under semi-lethal low light were investigated by RNA-Seq. The results were jointly analysed with morphological, cytomorphological and physiological observations in addition to gene expression analysis to understand cucumber seedling responses to low-light stress.

### 4.1 A low-light stress pressure at the semi-lethal time and morphological responses of the different cucumber lines tolerant to low-light stress

Low-light stress has adverse effect on plant growth and development, impairing several metabolic activities severely, and plants with different traits have different responses to low-light (Sekhar et al., 2019). The low light exposure having smaller of an effect on the photosynthetic process of the tolerant lines than sensitive lines, which due to the tolerant plant can optimize light capture though phenotypic plasticity (Valladares and Niinemets, 2008). To accurately clarify the characteristics of the response to low-light stress of both tested lines, a low-light treatment selection pressure at the semi-lethal time was proposed in this experiment. The semi-lethal time of plants under low-light treatment is the days when 50% of plants stopped growing or reached a half-dead state under low-light treatment. For example, the tolerant line M67 plants could live for 15 days before half of their growth stopped, and the sensitive line M14 plants stopped growing after 11 days of low-light treatment. Which due to the sensitive lines could not overcome the damage and became intolerant,under a longer duration of low-light treatment (Li et al., 2009).

The growth of cucumber seedlings involves the initiation of cell division and is rapidly occupied by newly dividing epidermal cells under normal-light conditions; however, the growth and metabolism of cucumber plants, especially sensitive lines, are inhibited under low light (Ying and Tai, 2020). Some researchers have reported that the morphological structure of plants has a certain initial adaptation to the growing environment, and low-light stress has an adverse effect on plant growth and development, impairing several metabolic activities (Hendry et al., 1987; Yao et al., 2007; Tian et al., 2017). Our findings also showed that the growth of cucumber seedlings began with the increase in internode length in the low-light-tolerant line with no terminal flowering (**Figure 2A**) compared to the slow growth of the low-light-sensitive line with terminal flowering (**Figure 2D**), which may have been caused by the slower cell elongation rate due to the weaker photosynthesis and self-protection of cucumber plants.

### 4.2 Leaf chloroplast ultrastructure and photosynthetic pigment responses of differently tolerant cucumber lines to low-light stress

The chloroplast ultrastructure of leaves under stress is an important indicator of resistance, and the thylakoid membrane system reacts dynamically to environmental cues, particularly light intensity and quality (e.g., Kirchhoff et al., 2004; Garab, 2016; Zhen and Zhang, 2000). The leaf anatomical structure and chloroplast ultrastructure of leaves at seedling stage were affected by low light stress, which varied in different tolerant cucumber plants. I.e., the palisade tissue cells become shorter and loosely arranged, the intercellular space of the spongy tissue increased, and the amount of spongy tissue decreased in the sensitive lines (Gao et al., 2009; Sheue et al., 2015). As Pribil et al. (2014) reported that the membrane structure was severely damaged and shape of the chloroplasts became into irregular rotund or oval under stress, our results also showed that the granum lamella in the chloroplast of sensitive lines was disintegrated and ruptured, meanwhile, the amount of starch grains decreased, the osmiophilic cells appeared at the same time (**Figure 5D**). As in previous studies, there were marked differences in chloroplast ultrastructure between the different tolerant lines, and the chloroplasts developed relatively easily, with more grana and closely arranged stromal thylakoids in tolerant lines (Zhen and Zhang, 2000), we found the similar results in tolerant line M67 (**Figure 5A**).

A previous study suggested that Chl content and the ratio of chlorophyll a to b (Chl a/b) in the leaves are dynamic to adapt to variations in light intensity to a certain degree, in theory determining the potential grain yield of plants (Zheng et al., 2012; Esteban et al., 2015). As the duration of stress increases, Chl a molecules experience increasingly severe damage, and the Chl a content decreases sharply; however, a high Chl b content and more photolytic Chl a/b protein complexes are beneficial to the absorption and utilization of light for plants (Ito et al., 1996; Yao et al., 2007). Notably, the extent of the increase was higher for Chl b than for Chl a, resulting in a reduced Chl a/b in response to low light, which indicates that the increase in Chl content in response to low light was mainly attributed to the enhanced production of Chl b (Zhang et al., 2014). We also found that the Chl a/b in the leaves of the tolerant line (M67) under low-light stress was lower than that under normal-light conditions, and the Chl a/b of the sensitive inbred line (M14) did not substantially change under either light condition (**Figure 6B**). Which may be due to the severe damage to chloroplasts caused by low-light stress, resulting in a sharp decrease in Chl content, especially the content of Chl b. The Chl content in cucumber seedlings increased significantly in the earlier stage of low-light stress; moreover, the content of Chl b increased more than that of chl a did in both treatments, and Chl a/b decreased (Liu et al., 2014). In the later part of the low-light stress, Chl was degraded rapidly, and the content of Chl in the sensitive line decreased significantly, which might be related to the severe damage to the chloroplasts in the leaves of the sensitive line. However, the Chl content in the tolerant line increased, bcause there was no big damage in leaves of tolerant line, so the tolerant plant could catch more light *via* increased Chl content, especially the Chl b content, in the leaves to adapt to the low-light stress environment (**Figure 6A**). Earlier research also indicated that, compared with sensitive lines, lines that are tolerant to low light contain higher Chl b in their leaves and maintain a lower Chl a/b when subjected to low light compared to normal light (Yan and Wang, 2013; Zhang et al., 2014).

### 4.3 Response of photosynthetic ability and photosynthate accumulation to the low-light stress in the different cucumber lines

Previous studies indicated that light limitation or shading stress decreased carbon fixation and canopy net photosynthetic rate and had negative effects on photosynthesis (Kromdijk et al., 2008; Gao et al., 2017). Low-light environments interfere with the normal photosynthetic activity of plants and reduce the accumulation of photosynthate by affecting starch and sucrose metabolism in the carbon source (Li et al., 2008; Vico et al., 2011). There was a sharply decreased rate of photosynthesis accumulation in the leaves of the sensitive line (M14) compared with those of the tolerant line (M67) (**Figures 3A, B**) and the line M67 presented consistent yields under normal as well as low-light conditions (Li et al., 2015). To explain differences in photosynthate accumulation in the different low-light-tolerant lines, the photosynthesis of cucumber seedling leaves was measured, and we found that there was no significant decrease in the tolerant line after low-light treatment; however, there was a decrease in Pn of more than 50% in the sensitive line (M14), and the photosynthesis capacity of the leaves of different low-light-tolerant lines varied after low-light treatment (**Figure 4**). We also found that there was no change for Tr of sensitive line seedlings under low-light conditions, but there was large increase in the tolerant line, which might be due to the decrease in Gs and Ci of functional leaves (**Figures 3B–D**). Researchers also revealed that the altered structures of the stomata, chloroplast lamellae and thylakoids under low-light stress resulted in a decreased concentration of CO_2_ and the rate of electron transfer in chloroplasts, thereby decreasing the ability of photosynthate accumulation, which might explain the decrease in the amount of starch grains in rice (Yamori et al., 2020). Our study revealed that the tolerance of M67 plants maintained their carbohydrate production levels by maintaining an efficient Pn even under low light, which in turn was achieved by maintaining higher levels of Chl content and photosynthetic enzyme activity compared to those maintained in the low-light-sensitive lines (**Figure 4**).

### 4.4 Transcriptome analysis and expression of the photosynthesis-related genes in different low-light tolerant cucumber lines

Photosynthesis was dramatically affected by low-light stress, and photosynthesis ability and photosynthate accumulation decreased under stress, which resulted in the reduced expression level of genes involved in photosynthesis (Zhang et al., 2017; Zeng et al., 2021). After the semi-low light treatment, the ATPase activity (**Figure 4**) and the expression levels of the *CsPsbQ* and *Csgamma genes* (**Figure 10B**) in cucumber seedling leaves were lower than those under normal-light conditions. Which were consistent with the earlier results that when photosystem was damaged during stress conditions, PSII repair and Chl turnover subsequently occur; more genes of PSII and genes encoding LHC were downregulated (Qiu et al., 2019). In this study, we conducted a comparative transcriptome profiling of low light tolerant and sensitive cucumber lines induced by low-light stress at seedling stage and found that some genes related to photosynthesis expressed at higher levels in the leaves of the low-light-tolerant line, but expressed at lower levels in leaves of the sensitive lines. Similar results were also reported in wheat in low tolerant cultivar of rice Swarnaprabha and low light sensitive rice cultivar IR8 (Sekhar et al., 2019). Which may be one of the main reason why the low-light-tolerance line was more tolerant to low-light stress.

*HEMA* and *HemY* are crucial genes involved in the early steps from the first committed precursor ALA to protoporphyrin IX in the tetrapyrrole biosynthetic pathway (Battersby, 2000; Tanaka et al., 2011). After low-light treatment, the expression of *CsHemA* and *CsHemH* were upregulated in tolerant line but the gene *CsHemY* and most of the genes related to Chl synthesis were downregulated, especially in the low-light-sensitive line (**Figures 8**, **9**), which in accordance with previous in cucumber result that the tetrapyrrol biosynthesis pathway downstream of ALA could redirect its focus to heme branch to adapt stress condition (Wu et al., 2018). In addition, chlorophyllide a oxygenase (*CAO*) is a key gene that controls how some of the newly synthesized Chl a or chlorophyllide a is converted into Chl b or chlorophyllide b (Tanaka et al., 1998; Espineda et al., 1999). The expression change of genes(i.e., *CsCAO、CsHCAR、CschL* and *Csnol*) related chlorophyll metabolism contributed to a suitable portion of Chl a/b being available for the tolerant cucumber plants to adapt to the low-light stress. After low-light treatment, the expression of *CschL* and *Csnol* increased in the leaves of the tolerant line, which indicated that the tolerant line could synthesize more Chl and maintain the suitable portion of Chl a/b to improve its photosynthetic capacity. Similar results have been reported in which *ZmCAO1* contributed to grain yield and waterlogging tolerance in maize (Li et al, 2021). We found that the expression of *CsHCAR* was downregulated in the leaves of the low-light-sensitive line. Recent studies have shown that the *CsHCAR* affects the stability of photosynthetic proteins in chloroplasts and positively regulates Chl degradation under different stresses, and the expression level of *CsHCAR* was the highest in senescent leaves of cucumber plants (Liu et al., 2021).

Genes encoding carbohydrate biosynthesis-related enzymes showed decreased transcript abundance under low-light stress, while carbohydrate degradation-related genes showed increased transcript abundance (**Figure 10C** and **Supplementary Table 17**). Except for the gene *CsBam6-3* (encoding glycoside hydrolase protein required for starch breakdown), which increased in both inbred lines after low-light treatment, the relative expression levels of mostly starch- and sucrose-synthesis genes decreased after low-light treatment. Under low-light stress, the total contents of sugar and starch in the leaves of the sensitive line were less than those under normal-light conditions, and those of the tolerant line were greater (**Figures 2**, **10C**). These results implied that the photosynthesis ability and glycosylation of the sensitive line were weaker under low-light stress, which may have been caused by destruction of the leaf structure and chloroplast ultrastructure. In short, we have elaborated the scientific phenomenon through which Chl synthesis is blocked and photosynthate accumulation is reduced under low-light treatment by identifying the genes related to Chl metabolism and glucose/starch metabolism.

## 5 Conclusion

Our research indicated that cucumber plants exhibited critical developmental changes at the critical semi-lethal stage under low-light stress. The photosynthetic capacity of leaves and photosynthate accumulation in cucumber plants were influenced by low light, which were mainly caused by the changes in the structure of leaves and chloroplasts, altered activity of photosynthesis-related enzymes and changes in gene expression levels, all of which differed between the low-light-tolerant and low-light-sensitive lines. Specifically, there was a sharply decreased rate of photosynthate accumulation in the leaves of the sensitive cucumber line, which was due to a reduced Chl content and disrupted chloroplast ultrastructure of the leaves, and the expression level of genes related to photosynthesis decreased under low-light stress. However, there was no significantly decreased or slight decrease in photosynthate accumulation or gene expression levels in the tolerant line. The transcriptome data also indicated that genes related to Chl synthesis and the starch- and sugar-metabolism pathways were differentially expressed in the different cucumber lines. In this study, the characteristics of plant phenotypes and gene expression changes in cucumber lines with different light tolerances under low-light stress were compared to determine the key period and change characteristics of cucumber plants in response to low-light stress. Further phenotypic and genotypic identification and functional studies are expected to screen the key genes related to response to low light, the findings of which will provide a reference for early screening of low-light-tolerant cucumber germplasm resources and low-light breeding.

## Data availability statement

The datasets presented in this study can be found in online repositories. The names of the repository/repositories and accession number(s) can be found in the article/**Supplementary Material**. The data presented in the study are deposited in the NCBI repository, accession number is PRJNA905893.

## Author contributions

DL and FY designed the experiment and secured the funding. FY, YZ, and KH conducted the experiment and data analysis, and FZ and RS created the figures. DD and YS performed the transcriptome data analyses. DD and FY wrote the manuscript. All authors contributed to the article and approved the submitted version.

## Supplementary material

Supplementary Figure 1 The index of low-light tolerance among different cucumber lines. Lowercase letters a and b after the value represent statistically significant differences (p < 0.05) within a variety under different treatments as determined by the least significant difference test.

Supplementary Figure 2 Effects on expression of genes related in photosynthesis of low-light stress. The **(A)** and **(B)** are each expression of genes encoding photosynthesis in M67 and M14 leaves; and the **(C)** and **(D)** are each expression of genes encoding light-harvesting complex II chlorophyll a/b binding proteins in M67 and M14 leaves.

Supplementary Figure 3 GO_enrich_Molecular_Function_enrich_dotplot of 932 DEGs in both lines (M67CK Vs M67T and M14CK Vs M14T).

Supplementary Figure 4 KEGG classification of 55 differential genes related to photosynthesis.

Supplementary Figure 5 GO_enrich_Biological_Process_enrich_dotplot of 932 DEGs in both lines (M67CK Vs M67T and M14CK Vs M14T).

Supplementary Figure 6 GO_enrich_Cellular_Component_enrich_dotplot of 932 DEGs in both lines (M67CK Vs M67T and M14CK Vs M14T).
