# Supplementary figures and images for: Integrative analysis of different low-light-tolerant cucumber lines in response to low-light stress
Source: Front Plant Sci. 2023 Jan 18;13:1093859. doi: 10.3389/fpls.2022.1093859 (PMC9891299; doi:10.3389/fpls.2022.1093859)

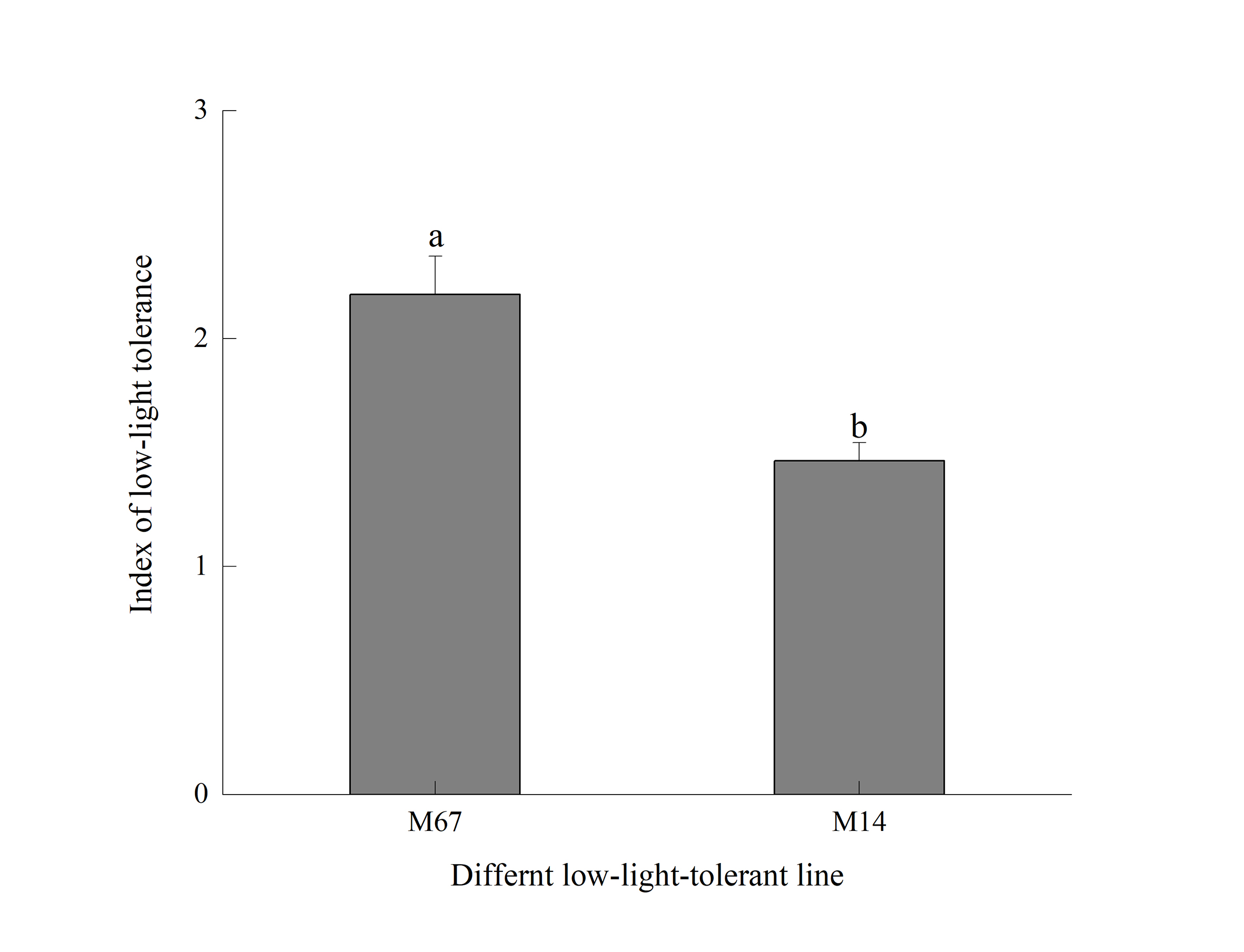

Supplement: Supplementary Figure 1 — The index of low-light tolerance among different cucumber lines. Lowercase letters a and b after the value represent statistically significant differences (p < 0.05) within a variety under different treatments as determined by the least significant difference test. [file Image_1.jpeg]

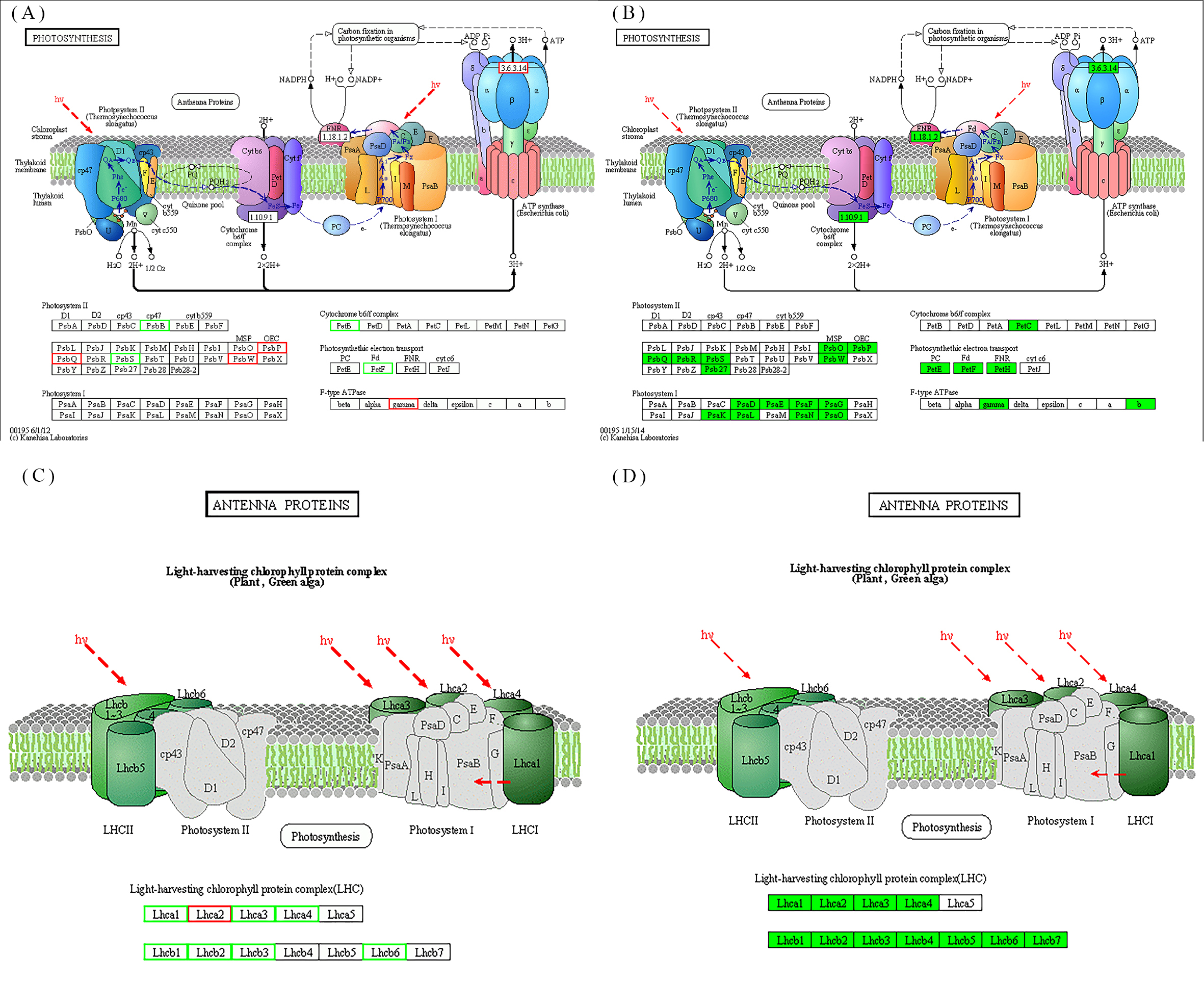

Supplement: Supplementary Figure 2 — Effects on expression of genes related in photosynthesis of low-light stress. The (A) and (B) are each expression of genes encoding photosynthesis in M67 and M14 leaves; and the (C) and (D) are each expression of genes encoding light-harvesting complex II chlorophyll a/b binding proteins in M67 and M14 leaves. [file Image_2.jpeg]

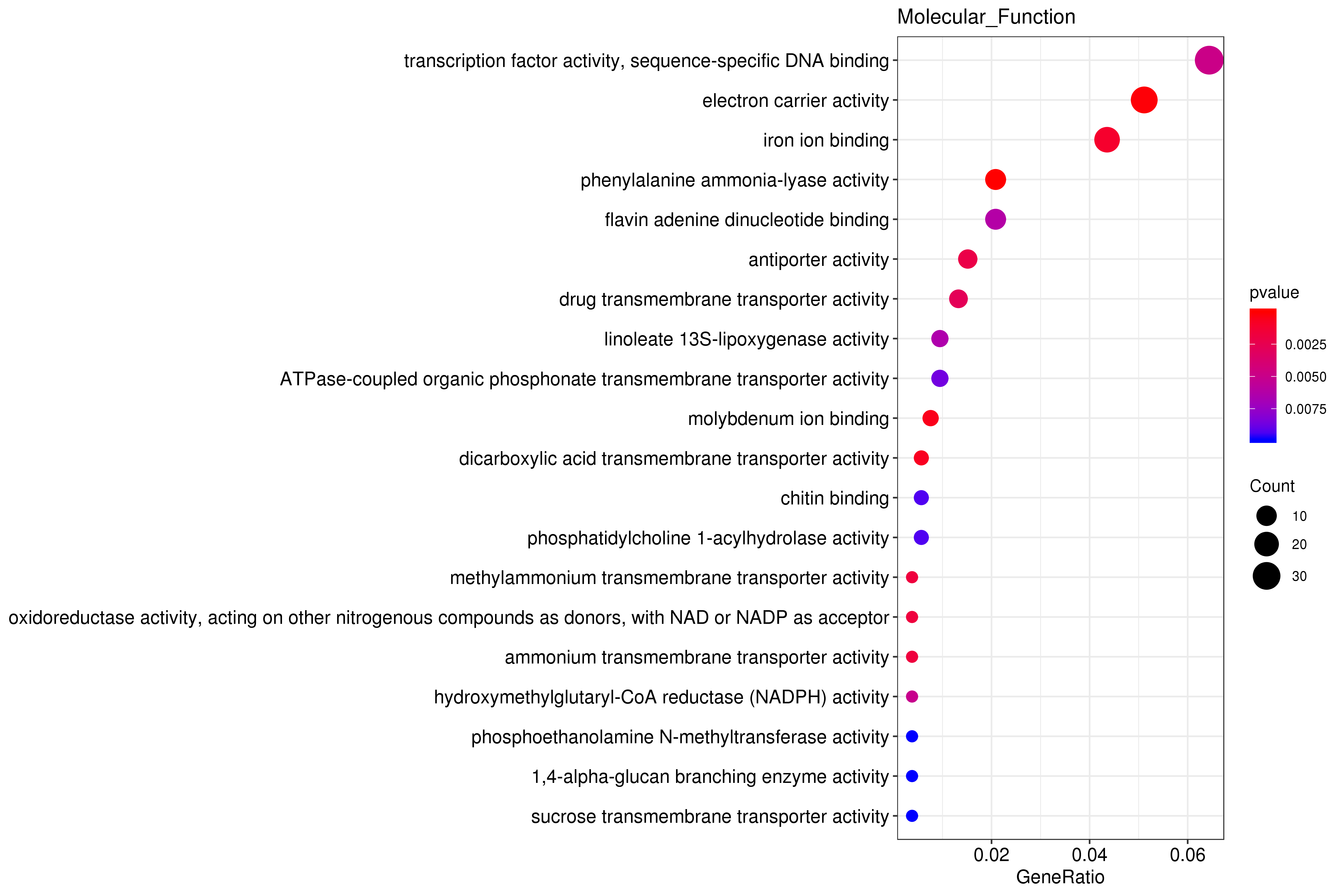

Supplement: Supplementary Figure 3 — GO_enrich_Molecular_Function_enrich_dotplot of 932 DEGs in both lines (M67CK Vs M67T and M14CK Vs M14T). [file Image_3.png]

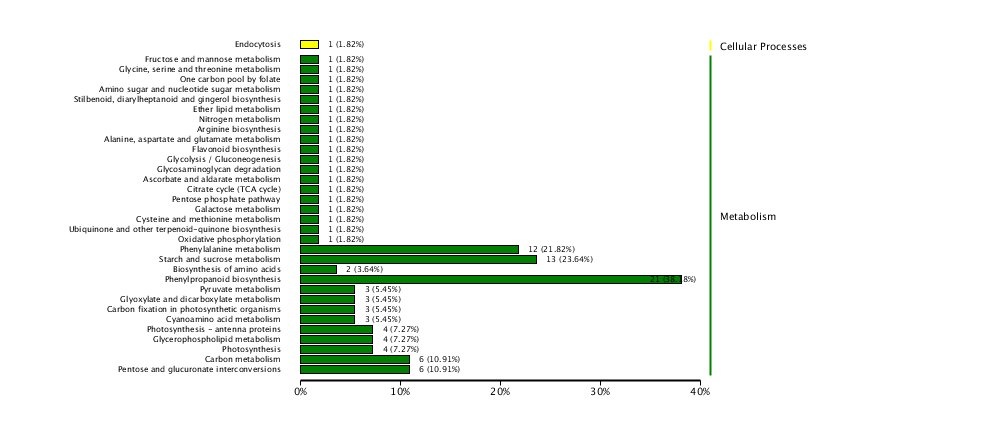

Supplement: Supplementary Figure 4 — KEGG classification of 55 differential genes related to photosynthesis. [file Image_4.png]

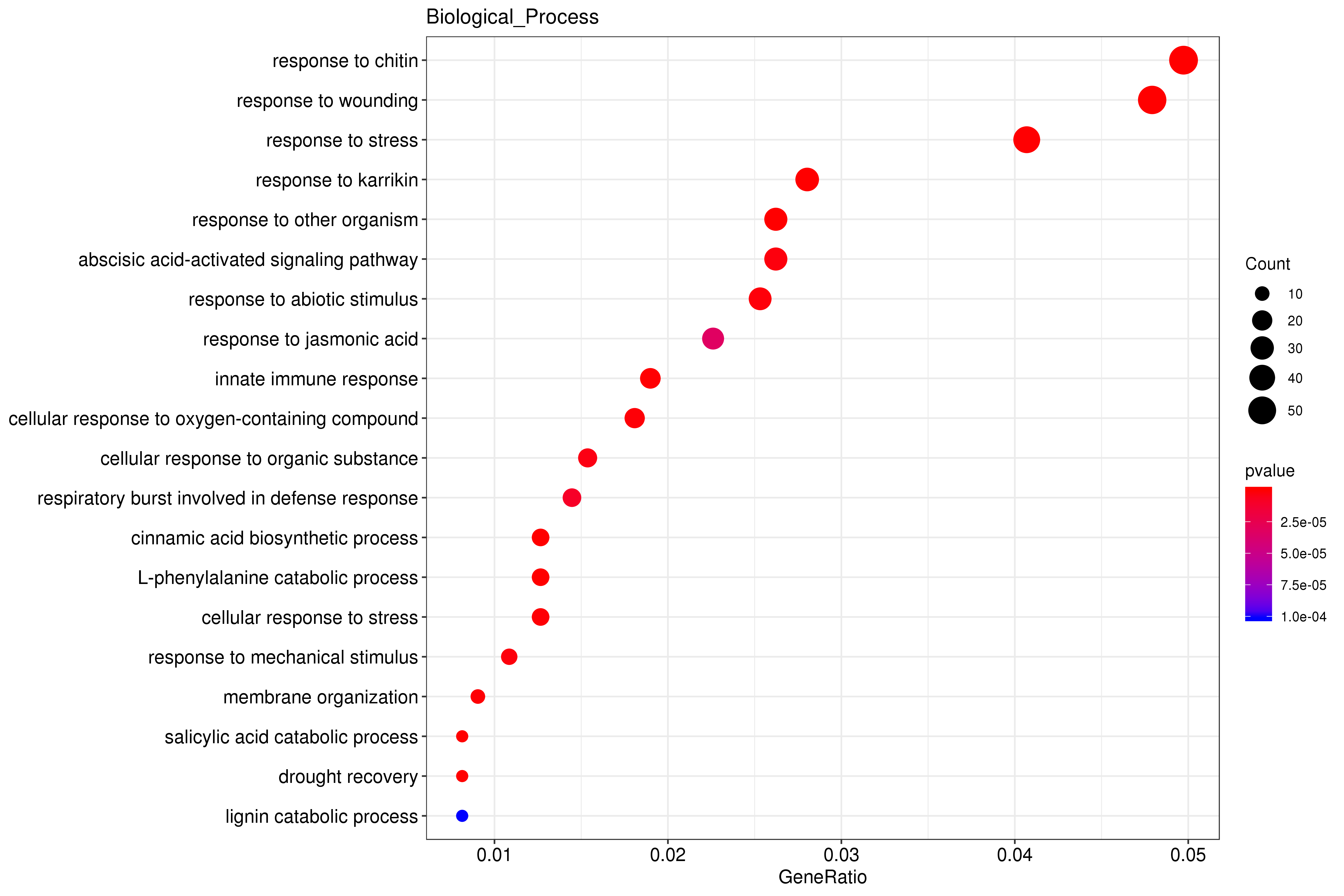

Supplement: Supplementary Figure 5 — GO_enrich_Biological_Process_enrich_dotplot of 932 DEGs in both lines (M67CK Vs M67T and M14CK Vs M14T). [file Image_5.png]

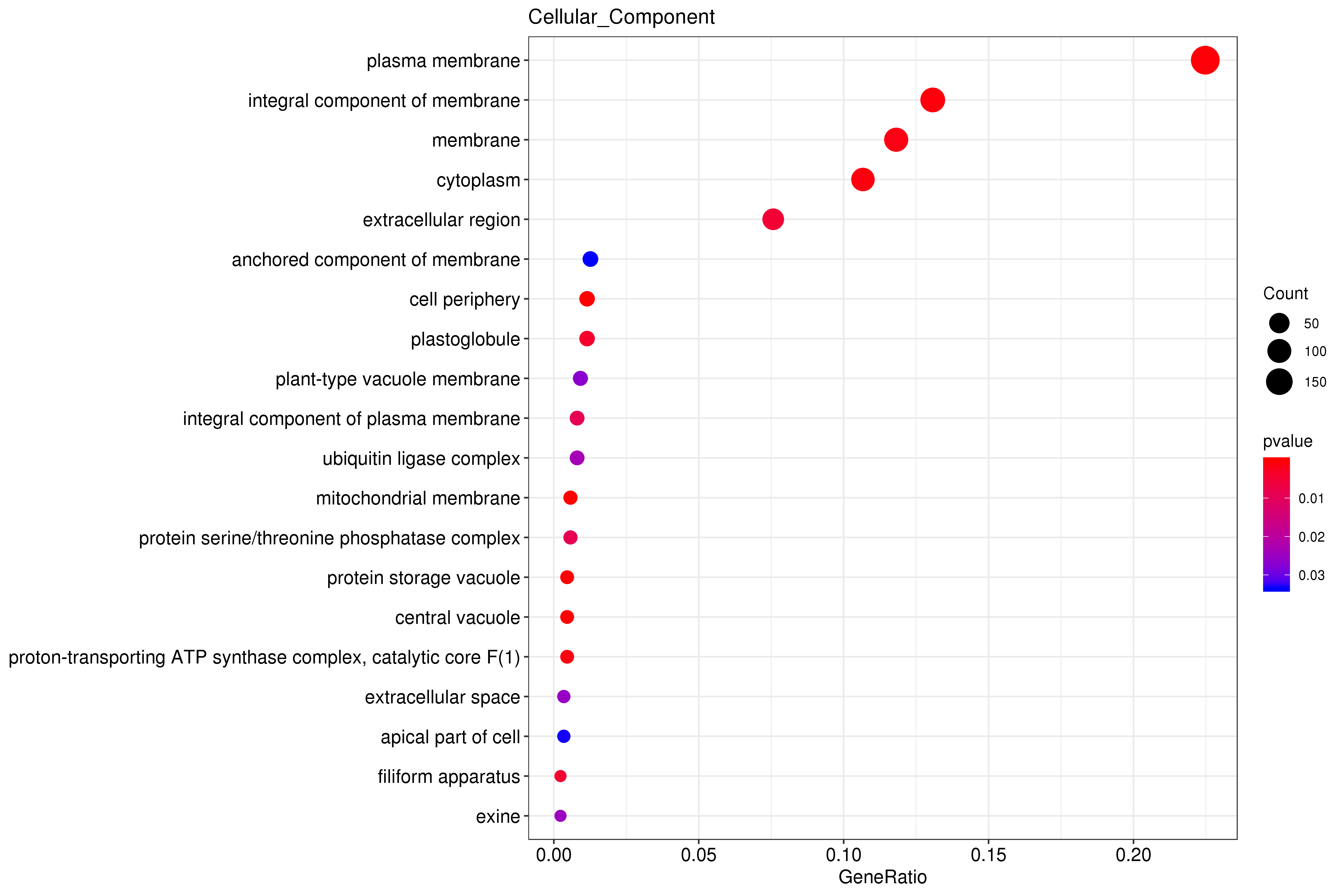

Supplement: Supplementary Figure 6 — GO_enrich_Cellular_Component_enrich_dotplot of 932 DEGs in both lines (M67CK Vs M67T and M14CK Vs M14T). [file Image_6.png]
