# Supplementary material for: Integrative analysis of different low-light-tolerant cucumber lines in response to low-light stress
Source: Front Plant Sci. 2023 Jan 18;13:1093859. doi: 10.3389/fpls.2022.1093859 (PMC9891299; doi:10.3389/fpls.2022.1093859)
Supplement: Supplementary file 7 [file DataSheet_1.docx]

Supplementary Material

# 1、Supplementary Tables

**Supplementary Table 1.** The index of low-light tolerance among different cucumber lines

**Supplementary Table 2.** Sucrose content in leaf under control and low-light stress in different cucumber lines

**Supplementary Table 3.** Starch content in leaf under control and low-light stress in different cucumber lines

**Supplementary Table 4.** Pn 、Gs、Ci and Tr of leaf under control and low-light stress in different cucumber lines

**Supplementary Table 5.** The ATPase activity of leaves in different low-light-tolerance cucumber lines

**Supplementary Table 6.** Chl (a+b) content in leaf of in different low-light-tolerance cucumber lines

**Supplementary Table 7.** The Chl a/b of leaf in different low-light-tolerance cucumber lines

**Supplementary Table 8.** Gross statistics of transcriptome sequencing alignments data

**Supplementary Table 9.** Statistics of differentially expressed genes in leaves of different lines under the control and low-light conditions

**Supplementary Table10-1.** The informations of EGG_pathway_enrich_dotplot

**Supplementary Table 10-2.** The KEGG classification of 55 differential expression genes related to photosynthesis

**Supplementary Table 11.** Statistics of photosynthesis and complex II chl a/b binding protein (LHC) DEGs expression

**Supplementary Table 12.** Statistics of porphyrin and chlorophyll metabolism DEGs expression

**Supplementary Table 13.** Statistics of starch and sucrose metabolism DEGs expression

**Supplementary Table 14.** The shared DEGs related with starch and sucrose pathway

**Supplementary Table 15.** Expression of the Chlorophyll synthesis related genes

**Supplementary Table 16.** Expression of the photosynthesis related genes

**Supplementary Table 17.** Expression of the starch and sucrose related genes

# Supplementary Figures

**Figure S1.** The index of low-light tolerance among different cucumber lines. Lowercase letters a and b after the value represent statistically significant differences (p < 0.05) within a variety under different treatments as determined by the least significant difference test.

**Figure S2.** Effects on expression of genes related in photosynthesis of low-light stress.The “(A)” and “(B)” are each expression of genes encoding photosynthesis in M67 and M14 leaves; and the “(C)” and “(D)” are each expression of genes encoding light-harvesting complex II chlorophyll a/b binding proteins in M67 and M14 leaves。

**Figure S3.**GO_enrich_Molecular_Function_enrich_dotplot

**Figure S4.** KEGG classification of 55 differential genes related to photosynthesis.

**Figure S5.**GO_enrich_Biological_Process_enrich_dotplot

**Figure S6.**GO_enrich_Cellular_Component_enrich_dotplot
